# The Spouses of Stroke Patients Have a Similar Oral Microbiome to Their Partners with an Elevated Risk of Stroke

**DOI:** 10.3390/microorganisms10112288

**Published:** 2022-11-18

**Authors:** Huidi Wang, Mengjia Yang, Sanping Cheng, Yueran Ren, Yiting Deng, Jingru Liang, Xiaofei Lin, Jie Li, Jia Yin, Qiheng Wu

**Affiliations:** 1 Department of Neurology, Nanfang Hospital, Southern Medical University, Guangzhou 510515, China; 2 Microbiome Medicine Center, Department of Laboratory Medicine, Zhujiang Hospital, Southern Medical University, Guangzhou 510515, China

**Keywords:** oral microbiome, stroke risk, spouses

## Abstract

Spousal members who share no genetic relatedness show similar oral microbiomes. Whether a shared microbiome increases the risk of cerebrovascular disease is challenging to investigate. The aim of this study was to compare the oral microbiota composition of poststroke patients, their partners, and controls and to compare the risk of stroke between partners of poststroke patients and controls. Forty-seven pairs of spouses and 34 control subjects were recruited for the study. Alcohol use, smoking, metabolic disease history, clinical test results, and oral health were documented. Oral microbiome samples were measured by 16S rRNA gene sequencing. The risk of stroke was measured by risk factor assessment (RFA) and the Framingham Stroke Profile (FSP). Poststroke patients and their partners exhibited higher alpha diversity than controls. Principal-coordinate analysis (PCoA) showed that poststroke patients share a more similar microbiota composition with their partners than controls. The differentially abundant microbial taxa among the 3 groups were identified by linear discriminant analysis effect size (LEfSe) analysis. The risk factor assessment indicated that partners of poststroke patients had a higher risk of stroke than controls. Spearman correlation analysis showed that *Prevotellaceae* was negatively associated with RFA. *Lactobacillales* was negatively associated with FSP, while *Campilobacterota* and *[Eubacterium]_nodatum*_group were positively associated with FSP. These results suggest that stroke risk may be transmissible between spouses through the oral microbiome, in which several bacteria might be involved in the pathogenesis of stroke.

## 1. Introduction

Stroke is the second most common cause of death globally and the most frequent cause of long-term disability in developed countries [1]. The risk factors for stroke can be categorized as nonmodifiable factors such as age, sex, and race, and modifiable factors such as smoking, alcohol consumption, hypertension, diabetes, and dyslipidemia [2]. Over the years, the gut microbiome has been identified as a potential risk factor for stroke. Indeed, patients with obesity, diabetes, and other metabolic diseases present dysbiotic gut microbiota compared to healthy controls [3]. Although not as substantially studied as the gut microbiota, the oral microbiota has been indicated to be more connected with the gut microbiota than previous research has suggested [4]. This association has given rise to concerns about the relationship between oral microbiota and stroke risk [5].

It is estimated that the human oral cavity harbors more than 700 kinds of microorganisms [6]. The oral microbiome represents one of the most important microbial communities in the human body and is included as one of the five research priorities of the human microbiome project (HMP), which includes the intestine, oral cavity, skin, vagina, and nasal cavity [7]. With the research development of the microbiome field, the link between the oral microbiome and a series of human chronic diseases has been revealed, such as cardiovascular disease [8], diabetes [9], high blood pressure [10], Alzheimer’s disease [11], cancers [12], and inflammatory bowel disease [13]. Oral microbiota dysbiosis is directly associated with the development of oral mucosal disease and periodontitis, which causes chronic arterial inflammation and increases the risk of cardiovascular diseases [14,15]. For example, *Fusobacterium nucleatum* and *Porphyromonas gingivalis* have been demonstrated to aggravate chronic inflammation [8]. The bacteria-derived metabolites, such as the lipopolysaccharide, recruit peripheral neutrophils that are elevated in the blood of atherosclerosis patients, indicating a role of endotoxin-mediated immunity in the progression of cardiovascular diseases [16]. In addition, periodontal microbiota dysbiosis also promotes insulin resistance, exacerbating the incidence and progression of diabetes [17]. Imbalances of oral commensal nitrate-reducing bacteria, including *Veillonella*, *Actinomyces*, *Haemophilus*, and *Neisseria*, have been associated with a decreased level of nitric oxide, causing endothelial dysfunction, and eventually lead to hypertension [18]. On the other hand, these inflammatory diseases can also modify oral microbiota composition possibly through enhanced inflammation, in which interleukin-17 may be the key mediator [19]. It has been shown that oral dysbiosis is associated with reduced amyloid beta 42 in the cerebrospinal fluid, but the underlying mechanism is unknown [11]. As for the role of oral microbiota in the development of cancer, it has been suggested that some oral microbiota, such as *Porphyromonas gingivalis* and *Fusobacterium nucleatum*, can promote cancer growth by inhibiting apoptosis, activating cell proliferation, promoting cellular invasion, and directly producing carcinogens [12]. It has also been proposed by some researchers that oral diseased-related bacteria directly exacerbate inflammatory bowel disease through translocating from the oral cavity to the intestine [13]. It has been suggested that the oral microbiome can reflect human health and disease status in a real-time manner and has important value in the early warning of disease risk [20].

The past century has seen a remarkable decrease in mortality rates across the globe, with a shift from communicable diseases (infectious diseases) to noncommunicable diseases (metabolic diseases, cancers, etc.). It has been suggested by some researchers that the microbiota is involved in these noncommunicable diseases [21]. Expectedly, as family members share similar environments and diets, their microbiota tend to be similar. Interestingly, cohabitants and spouses have more similar gut microbiota than their siblings living separately even though they share similar a genetic background [21]. Compared to other family members, spouses may have a more similar oral microbiome because of some intimate behaviors, such as kissing. It has been reported that partners have a more similar oral microbiota composition than do unrelated individuals, and interestingly, there is an average of 80 million bacterial transfers per 10 s of intimate kissing between partners [22].

This study aimed to investigate the oral microbiome of poststroke patients and their partners, and compared their oral microbiome with those of controls without stroke history (including their partners). Furthermore, we aimed to evaluate the risk factors for stroke between partners of stroke patients and controls, and identify the microbiome that is associated with stroke risks.

## 2. Materials and Methods

### 2.1. Ethics Statement and Participants

All participants provided written informed consent following the rules of the Declaration of Helsinki. This study was approved by the Ethics Committee of Nanfang Hospital, Southern Medical University (NFEC-2020-169) and registered at http://clinicaltrials.gov (accessed on 2 August 2021) (NCT04688138). Stroke patients were recruited from the Department of Neurology of Nanfang Hospital of Southern Medical University (Guangzhou, China) from June 2020 to April 2021. The oral samples were collected 3 months after a stroke. This timepoint was chosen based on the notion that the microbiota changes rapidly and dynamically during the acute stage of stroke, while it stabilizes during the recovery stage. The inclusion criteria were as follows: (i) a diagnosis of ischemic stroke without thrombolytic therapy or thrombectomy, (ii) a National Institutes of Health Stroke Scale (NIHSS) score ≤ 15, and (iii) an age greater than 18 years. The control subjects were recruited from the community without a history of stroke (including their partners). The absence of a history of stroke was confirmed by inquiring about past medical history and checking the medical systems if the participants had been admitted to the hospital. The exclusion criteria for all participants were as follows: (i) patients who were administered antibiotics or probiotics within 3 months before admission or during follow-up; (ii) patients with severe liver diseases, renal diseases, oral diseases, gastrointestinal diseases, or infectious diseases; and (iii) patients from whom samples could not be obtained during follow-up.

### 2.2. Clinical Data and Sample Collection

The clinical data and medical history were collected by trained neurologists from Nanfang Hospital of Southern Medical University. The risk factor assessment for stroke was conducted as we previously reported [23]. Briefly, the assessment included hypertension, diabetes mellitus, dyslipidemias, atrial fibrillation, overweight, smoking, physical inactivity, and family history of stroke [24], following the protocols formulated by the National Health and Family Planning Commission of Stroke Screening and Prevention Project. The oral and blood samples of stroke patients and their partners were collected at the 3-month follow-up visit after stroke onset. Fasting blood samples were examined in the Clinical Laboratory of Nanfang Hospital, and routine blood parameters were estimated. After fasting overnight, participants were asked to wash the oral cavity with clean drinking water, after which the subgingival surfaces of healthy sites were swabbed by a trained dentist with a sterile cotton swab and placed in an Eppendorf tube with 1 mL phosphate-buffered saline and swiftly transported and stored at −80 °C.

### 2.3. 16S RNA Sequencing and Analysis

Bacterial genomic DNA was extracted using a QIAamp Pro DNA Kit (QIAGEN, Valencia, CA, USA) according to the manufacturer’s instructions. The barcoded primers V4F (GTGYCAGCMGCCGCGGTAA) and V4R (GGACTACNVGGGTWTCTAAT) were used to amplify the V4 variable region of the 16S rRNA gene. PCR was performed according to a previously described method [25]. All PCR amplicons were mixed and sequenced using the Illumina iSeq 100 platforms. The Shannon index, phylogenetic diversity (PD) whole-tree index, Simpson index, and Chao1 index were determined to assess α-diversity. The Shannon and Simpson indexes indicate the number and distribution of microbial species in a sample. The observed species index indicates the number of species in the sample. The Chao1 index indicates community richness. Abund Jaccard, binary Jaccard, Bray-Curtis, Euclidean, and Kulczynski index plots were used to analyze the β-diversity by illustrating the phylogenetic dissimilarity among samples. A smaller distance between two samples indicates a higher similarity. As a dimensionality reduction method, principal-coordinate analysis (PCoA) was used to describe the relationships among samples based on the distance matrix and visualize the unsupervised grouping pattern of the complex data set, i.e., the microbiome. Linear discriminant analysis effect size (LEfSe) was used to compare the discriminative data between groups. As an algorithm for high-dimensional biomarker discovery, LEfSe identifies genomic features that characterize differences between two or more biological conditions. By emphasizing statistical significance, biological consistency, and effect relevance, LEfSe determines the abundant feature with the greatest difference between conditions in accordance with biologically meaningful categories. For each differential feature detected using LEfSe, we calculated a linear discriminant analysis value, representing the difference in the feature between groups.

### 2.4. Stroke Risk Evaluation

The risk factor assessment (RFA) has previously been reported [23]. Generally, RFA includes hypertension, diabetes mellitus, dyslipidemia, atrial fibrillation, overweight, smoking, physical inactivity, and a family history of stroke. The Framingham Stroke Profile (FSP) has also been widely used [26]; the assessment uses a Cox proportional hazards model with risk factors as covariates and points calculated according to the weight of the model coefficients (Supplementary Table S1).

### 2.5. Statistical Analyses

Data were analyzed using IBM’s SPSS 25.0 statistical software package. We used the mean (standard deviation) to express data that obeyed a normal distribution, the median (interquartile range) to express measurement data that obeyed a skewed distribution, and a percentage to express enumeration data. The normality of data distribution was checked with the Shapiro–Wilk test. Nonparametric tests (Kruskal–Wallis one-way ANOVA by ranks, or Mann–Whitney U test) were used to compare differences among the three groups, as appropriate. Spearman correlation analysis was used to analyze the correlations between the oral microbiota and stroke risk. Because the microbiome data are multidimensional, we used the Adonis test implemented in QIIME 1.9.0, which partitions a distance matrix among sources of variation to describe the strength and significance that a categorical or continuous variable has in determining the variation of distances. A value of *p* < 0.05 was considered statistically significant in the compared groups.

## 3. Results

### 3.1. Clinical Characteristics of the Study Population

**Figure 1:**
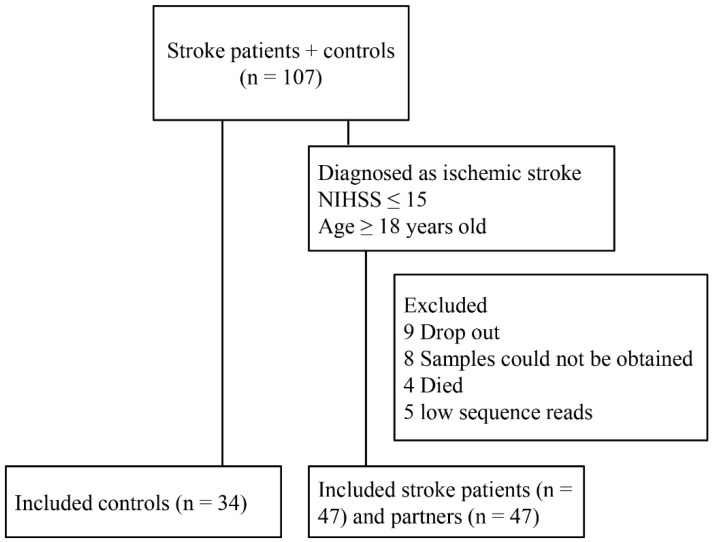
Flowchart of the study. In the beginning when the stroke patients were hospitalized, 73 patients were recruited for our study. At the 3-month follow-up, 9 patients dropped out of the study, samples from 8 patients could not be obtained, 4 patients died, and 5 samples were excluded due to low sequence reads. A total of 128 participants were recruited for the study, including 47 stroke patients and their respective partners and 34 controls.

In the beginning when the stroke patients were hospitalized, 73 patients were recruited for our study. At the 3-month follow-up, 9 patients dropped out of the study, samples from 8 patients could not be obtained, 4 patients died, and 5 samples were excluded due to low sequence reads. A total of 128 participants were recruited for the study, including 47 stroke patients and their respective partners and 34 controls (Figure 1). Demographic and clinical data for the participants are presented in Table 1. The mean age of poststroke patients, partners, and controls was 53.9, 52.7, and 56.7, respectively. The percentage of males was lower in the partners group than in the other groups. Furthermore, the percentages of smoking, alcohol consumption, hypertension, diabetes, hyperlipidemia, and hyperuricemia were higher in poststroke patients than in other groups. There were no significant differences in the percentage of caries, residual root, periodontitis or calculus between the three groups. For blood tests, the partner group had higher levels of platelets (PLT), albumin (ALB), and high-density lipoprotein cholesterol (HDL-C) and lower levels of neutrophils (NEU), cystatin C (Cys-C), direct bilirubin (DBIL), and glycosylated hemoglobin (HbA1c) than the control group (Table 2).

### 3.2. Comparisons of Microbial Alpha- and Beta-Diversity

**Figure 2:**
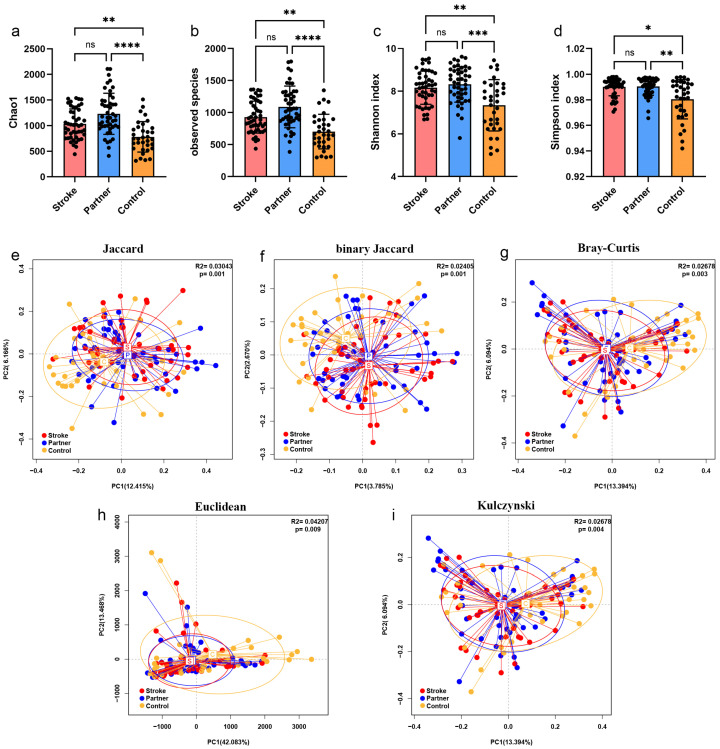
Comparisons of microbial alpha- and beta-diversity between the three groups. (**a**–**d**) Comparison of alpha-diversity index Chao1, observed species, Shannon index and Simpson index between the three groups. The Shannon and Simpson indexes indicate the number and distribution of microbial species in a sample. The observed species index indicates the number of species in the sample. The Chao1 index indicates community richness. (**e**–**i**) Comparison of beta-diversity index abund Jaccard, binary Jaccard, Bray-Curtis, Euclidean, and Kulczynski index between the three groups. A smaller distance between two samples indicates a higher similarity. As a dimensionality reduction method, principal-coordinate analysis (PCoA) was used to describe the relationships among samples based on the distance matrix and visualize the unsupervised grouping pattern of the microbiome. Data were expressed as mean ± SD, * *p* < 0.05, ** *p* < 0.01, *** *p* < 0.001, **** *p* < 0.0001. ns, no significance.

The Shannon index, PD whole-tree index, Simpson index, and Chao1 index were determined to assess α-diversity. The Shannon and Simpson indexes indicate the number and distribution of microbial species in a sample. The PD whole-tree index indicates the range of phylogenetic distances among microbial species. The Chao1 index indicates community richness. Based on alpha-diversity indexes, including Chao1, observed species, Shannon index and Simpson index, the control group showed lower diversity than the other groups (Figure 2a–d). To evaluate the compositional differences between the groups, beta diversity indexes, including the Jaccard, binary Jaccard, Bray-Curtis, Euclidean, and Kulczynski indexes were measured. Samples of the partner group were clustered closely to those of the stroke group, whereas samples of the control group were clustered separately from those of the stroke group and partner group (Figure 2e–i), indicating that poststroke patients share a more similar oral microbiota composition with their partners than controls.

### 3.3. Comparison of Microbial Composition

**Figure 3:**
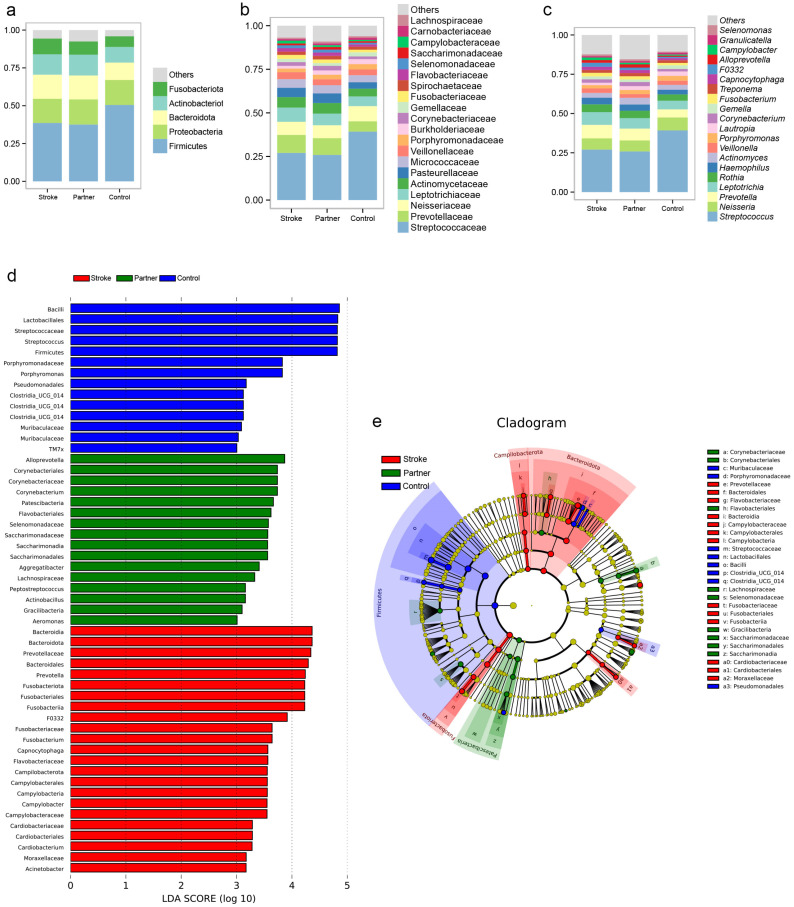
The oral microbiota composition and the significantly different taxa identified by LEfSe analysis among groups. (**a**–**c**) Stacked bar plot representing the relative abundances of microbiota at phylum, family, and genus levels, respectively. (**d**) A histogram of the linear discriminant analysis (LDA) plot performed by LefSe analysis shows distinct oral microbiome composition between the three groups. Red indicates enriched taxa in poststroke patients, green indicates the taxa enriched in partners of poststroke patients and blue indicates taxa enriched in the control group. (**e**) A cladogram for taxonomic representation performed by LefSe analysis showing distinct bacterial taxa between the three groups. As an algorithm for high-dimensional biomarker discovery, LEfSe identifies genomic features that characterize differences between two or more biological conditions. By emphasizing statistical significance, biological consistency, and effect relevance, LEfSe determines the abundant feature with the greatest difference between conditions in accordance with biologically meaningful categories. For each differential feature detected using LEfSe, a linear discriminant analysis value was calculated, representing the difference in the feature between groups. Only taxas with LDA scores above ±3.0 are shown.

**Figure 4:**
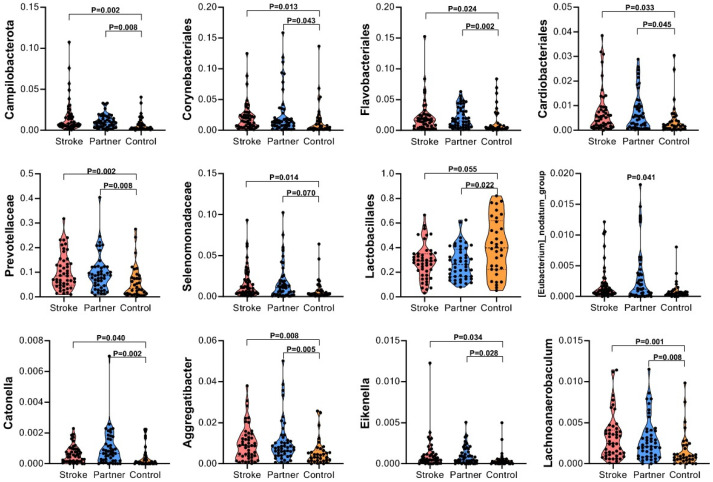
Comparison of several bacteria with different abundances among the three groups, i.e., *Campilobacterota*, *Corynebateriales*, *Flavobateriales*, *Cardiobacteriales*, *Prevotellaceae*, *Selenomonadaceae*, *Lactobacillales*, *[Eubacterium]_nodatum*_group, *Catonella*, *Aggregatibacter*, *Eikenella,* and *Lachnoanaerobaculum*, were calculated.

At the phylum level, the control group had a higher abundance of Firmicutes than the other groups (Figure 3a). At the family level, the control group had higher abundances of *Streptococcaceae* and *Neisseriaceae* and lower abundances of *Prevotellaceae*, *Leptotrichiaceae*, and *Actinomycetaceae* (Figure 3b) than the other groups. At the genus level, the control group had higher abundances of Streptococcus and Neisseria, and lower abundances of *Prevotella* and *Leptotrichia* than the other groups (Figure 3c). To identify microbial taxa with significantly different abundances between the three groups, linear discriminant analysis effect size (LefSe) analysis was performed with an LDA value of 3.0 (Figure 3d,e). In the stroke group, the differentially abundant taxa were *Bacteroidia*, *Prevotellaceae*, *Fusobacteriota*, *Campilobacterota*, *Cardiobacteriaceae*, etc. The differentially abundant taxa in the partner group were *Alloprevotella*, *Corynebacteriales*, *Patescibacteria*, *Flavobacteriales*, *Selenomonadaceae*, *Saccharimonadaceae*, etc. The differentially abundant taxa in the control group were *Bacilli*, *Lactobacillales*, *Streptococcaceae*, *Streptococcus*, etc. The comparison of bacteria between the 3 groups is presented in Figure 4.

### 3.4. Comparison of Stroke Risk

**Figure 5:**
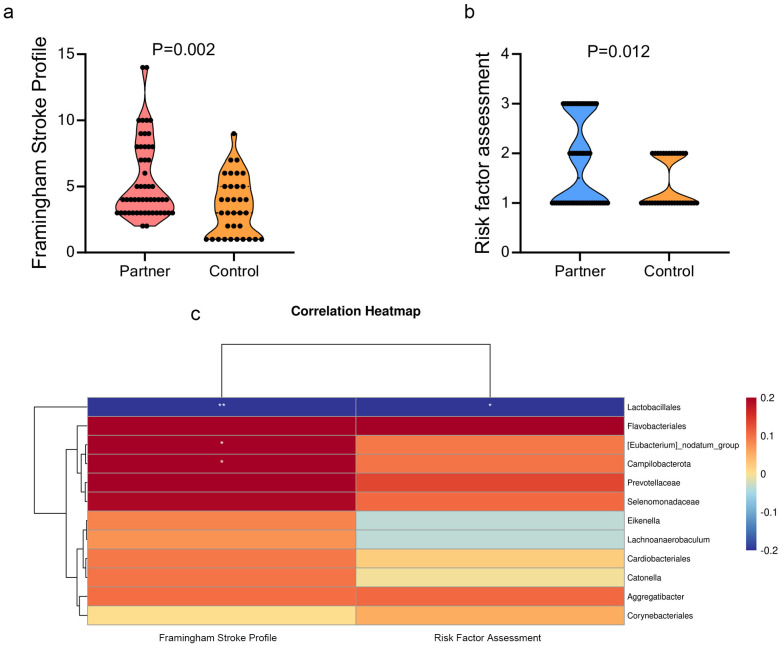
Stroke risk assessment between partners of poststroke patients and controls. (**a**,**b**) Comparison of Framingham Stroke Profile (FSP) and risk factor assessment (RFA) between the two groups. RFA includes hypertension, diabetes mellitus, dyslipidemia, atrial fibrillation, being over-weight, smoking, physical inactivity, and family history of stroke. The FSP uses a Cox proportional hazards model with risk factors as covariates and points calculated according to the weight of the model coefficients (Supplementary Table S1). (**c**) The association between oral microbiota and stroke risk. Heatmap of the Spearman’s correlation between discriminatory bacteria and stroke risk. Red squares indicate positive correlations, whereas blue squares indicate negative correlations. Lactobacillales was negatively associated with FSP. *Prevotellaceae* was negatively associated with RFA. *Campilobacterota* and *[Eubacterium]_nodatum*_group were positively associated with FSP. * *p* < 0.05, ** *p* < 0.01.

The partner group had a significantly higher Framingham Stroke Profile (FSP) score than the control group (Figure 5a). Additionally, scores of the risk factor assessment (RFA) were significantly higher in the partner group than in the control group (Figure 5b). To identify the oral microbiota that are associated with increased stroke risk, Spearman correlation analysis was used. *Lactobacillales* was negatively associated with FSP. *Prevotellaceae* was negatively associated with RFA. *Campilobacterota* and *[Eubacterium]_nodatum*_group were positively associated with FSP (Figure 5c).

## 4. Discussion

To the best of our knowledge, this is the first study to assess the oral microbiome of poststroke patients and their partners. In this study, we found that poststroke patients had a similar oral microbiome to their partners. Importantly, compared with controls whose partners did not have a history of stroke, spouses of stroke patients had an elevated risk of stroke, indicating that transmission of oral microbiota might be involved in the occurrence of stroke.

Several chronic diseases predispose people to stroke, including diabetes mellitus, hypertension, obesity, high cholesterol, etc. [27]. Genetics may play an important role in the pathogenesis of these chronic diseases [2]. Most epidemiologic investigations and public health interventions currently targeting these metabolic syndromes are focused on individuals or occasionally the community, while neglecting the family unit [28]. The household is potentially an important but understudied research field and a plausible target of intervention. The bulk of family-based studies have been conducted to understand the genetic predisposition to certain diseases. Unlike genetically related household members, such as parent-child or sibling pairs, spouses do not have similar genetic backgrounds. In our study, we found that spouses of stroke patients had an elevated risk of stroke compared with those with a partner who did not have a stroke history. Our findings are consistent with prior studies investigating the relationship of metabolic status among spouses [28,29,30,31]. Previously, a study conducted by Patel et al. [28] in India reported that adults who lived with another adult with any chronic diseases such as diabetes, hypertension, hyperlipemia and mental disorder, had higher odds of having one or more chronic diseases themselves. Moreover, a study conducted in Korea also found significant spousal concordance of metabolic syndrome [29], indicating shared environmental factors contributing to the development of metabolic diseases. However, how these conventionally considered noncommunicable diseases become communicable between spouses is unknown. Previous studies have suggested that assortative mating, health behavior, social factors, etc., account for similarities between spouses, but the underlying mechanism remains unknown. In this study, we found that the oral microbiota composition of poststroke patients resembles that of their spouses, which might contribute to the higher stroke risk. A similar oral microbiome between spouses may mediate the crosstalk between external factors such as dietary intake, lifestyle, environment, physical activities, intimate behaviors and similarities in stroke risk.

Although we could not determine whether the elevated stroke risk seen in spouses with stroke partners causally resulted from the oral microbiota, numerous studies have confirmed the role of the oral microbiota in the pathogenesis of metabolic or cardiovascular diseases [5,32,33,34,35]. According to current consensus, the hypothesis is that oral microbiota dysbiosis causes local infection and inflammation, and translocation of lipopolysaccharide in the circulation leads to chronic low-grade inflammation in the periphery, which contributes to metabolic dysregulation [34]. In the current study, oral status, such as dental caries, removable protheses and periodontal diseases, was not different between stroke partners and controls, so the elevated stroke risk in stroke partners might not be attributed to oral diseases. We observed higher abundances of *Neisseria*, *Fusobacterium*, *Capnocytophaga*, *Actinomyces*, *Eikenella* and *Selenomonas* in poststroke patients and their spouses than in controls, which is consistent with previous studies that found higher levels of the above oral microbiota in diabetic patients than in nondiabetic patients [36,37], suggesting that these bacteria may be associated with the etiology of diabetes, a well-known risk factor for stroke. However, inconsistently, the abundance of *Streptococcus* was higher in controls than in the other groups, thus warranting further investigation with a larger sample size in the future. Notably, oral microbiota, including *Neisseria*, *Fusobacterium*, *Streptococcus* and *Veillonella*, can be detected in atherosclerotic plaques [38,39,40,41], which are responsible for the occurrence of ischemic stroke and myocardial infarction. Moreover, the levels of *Neisseria*, *Fusobacterium* and *Streptococcus* were found to be associated with risk factors for stroke, such as blood cholesterol levels [38]. These findings indirectly support the possibility that oral microbiota travel through the oral mucosa to the peripheral blood vessel, which triggers a systemic immune response. In addition, the intestinal microbiota is also transmissible among family members particularly couples [42]. The intestinal microbiota has been widely studied as an important participant in the etiology of cardiovascular and metabolic diseases [43,44]. In addition, the oral microbiota can travel through the digestive tract and influence the composition of the gut microbiota [45]. Therefore, it is possible that the oral microbiota participates in the pathophysiology of stroke-related diseases by modulating the intestinal microbiota.

In this study, we found that *Campilobacterota* and *[Eubacterium]_nodatum*_group were positively associated with stroke risk. *Campilobacterota* are proinflammatory bacteria that are involved in sulfide-oxidizing processes and the biodegradation of polycyclic aromatic hydrocarbons [46,47,48]. *Eubacterium nodatum* within subgingival plaque is associated with periodontitis [49,50]. Interestingly, in a study with a cohort of 6491 participants, researchers found that periodontal antibodies against *Eubacterium nodatum* were associated with cancer mortality, representing a novel predictor of cancer mortality [51]. The abundance of intestinal *Eubacterium nodatum* is increased after a proinflammatory diet [52]. In addition, *Eubacterium nodatum* is enriched in the intestine after cerebral ischemic stroke and is considered a pathogen and an opportunistic microorganism [53]. However, whether *Eubacterium nodatum* originates from the oral cavity remains unknown and further investigations are needed to study the effects of these microbiota on the pathogenesis of metabolic and cardiovascular diseases.

We acknowledge several limitations of the current study. First, this is a cross-sectional study design, preventing any causal relationship between oral microbiota transmission and increased risk of stroke. A prospective cohort study to investigate the oral microbiome of spouses before and after the stroke event is needed to further investigate the causal relationship. Second, information concerning the socioeconomic status and household environment conditions was missing in this study, which affects the oral microbiome and thus might influence the co-occurrence of the oral microbiome. Third, the number of participants recruited in our study was small, and future studies with a larger sample size are warranted. Fourth, the baseline imbalance (including sex, smoking, drinking, and hypertension) between groups influenced the oral microbiota, which affected the credibility of the statistical results.

## 5. Conclusions

In conclusion, the current study demonstrated that spouses of poststroke patients exhibited oral microbiota compositions similar to those of their partner’s and had a higher risk of stroke than controls. The increased stroke risk seen in spouses of poststroke patients might be associated with oral microbiota, i.e., *Campilobacterota* and *[Eubacterium]_nodatum*_group. These findings might provide microbial biomarkers for the prediction of the risk of stroke in patients’ spouses.

## Figures and Tables

**Table 1:** Characteristics of the study participants.

		Stroke (*n* = 47)	Partner (*n* = 47)	Control (*n* = 34)	*p*
Age		53.9 ± 8.8	52.7 ± 9.3	57.0 ± 8.8	0.058
Sex (male)		39, 83.0% ^#^	8, 17.0% ^&^	21, 61.8%	0.001 *
Smoke		26, 55.3% ^#&^	3, 6.4%	5, 15.6%	0.001 *
Drink		22, 46.8% ^#&^	5, 10.6%	4, 12.5%	0.001 *
Hypertension		32, 68.1% ^#&^	18, 38.3%	12, 35.3%	0.003 *
Diabetes mellitus		15, 31.9%	9, 19.1%	6, 17.6%	0.226
Hyperlipoidemia		30, 63.8%	19, 40.4%	13, 38.2%	0.030 *
Hyperuricemia		17, 36.2%	13, 27.7%	11, 32.4%	0.678
Atrial fibrillation		0, 0%	0, 0%	0, 0%	1.000
Coronary atherosclerotic heart disease		3, 6.4%	0, 0%	1, 3.1%	0.208
Oral hygiene status	Caries experience	28, 59.6%	24, 51.1%	18, 52.2%	0.508
	Residual root	5, 10.6%	5, 10.6%	4, 11.8%	0.595
	Periodontal status (serious)	24, 51.1%	28, 59.6%	21, 61.8%	0.183
	Calculus (serious)	37, 78.7%	40, 85.1%	25, 73.5%	0.361
	Moveable denture	0, 0%	0, 0%	0, 0%	1.000
Dietary habit	Partial vegetarian	7, 14.9%	5, 10.6%	5, 14.7%	0.800
	Balance portion of vegetables and meat	33, 70.2%	34, 72.3%	19, 55.9%	0.258
	Partial predator	7, 14.9%	8, 17.0%	10, 29.4%	0.232

Data are presented as mean ± SD, or *n* (proportion). * *p* < 0.05, ^#^ *p* < 0.05 compare with the Partner group, ^&^ *p* < 0.05 compare with the Control group.

**Table 2:** Clinical test results of the participants.

	Stroke (*n* = 47)	Partner (*n* = 47)	Control (*n* = 34)	*p*
WBC, ×10^9^	7.09 (6.09–7.92)	6.32 (5.22–7.45)	6.97 (5.58–7.83)	0.091
HGB, ×10^9^	141.78 ± 2.38	136.19 ± 2.50	133.79 ± 2.65	0.101
NEU%	62.80 (52.40–71.40) ^&^	58.60 (53.95–632.75) ^&^	62.50 (54.59–65.17)	0.001 *
PLT, ×10^9^	213.0 (198.0–254.0) ^#^	261.0 (213.5–305.5) ^&^	205.5 (184.0–250.0)	0.001 *
UREA, μmol/L	5.2 (4.4–5.9)	5.0 (3.9–5.8)	4.7 (4.1–5.8)	0.680
UA, μmol/L	374.0 (307.0–431.0) ^#^	321.0 (292.0–372.0)	357.0 (323.0–405.0)	0.016 *
CR, μmol/L	78.0 (71.0–94.0) ^#^	64.0 (56.5–74.0) ^&^	73.5 (64.0–86.0)	0.001 *
Cys–C, mg/L	1.03 ± 0.06 ^#^	0.81 ± 0.03 ^&^	1.03 ± 0.06	0.001 *
ALT, U/L	29.02 ± 2.77 ^#&^	20.21 ± 2.22	18.55 ± 2.26	0.001 *
ALB, g/L	44.3 (42.2–47.1) ^&^	44.0 (42.9–45.4) ^&^	39.8 (38.0–42.0)	0.001 *
DBIL, μmol/L	4.24 ± 0.24 ^#^	3.07 ± 0.17 ^&^	5.03 ± 0.56	0.001 *
TG, mmol/L	1.22 ± 0.12 ^&^	1.59 ± 0.18	1.59 ± 0.13	0.025 *
CHOL, mmol/L	3.56 (3.24–3.97) ^#&^	5.32 (4.64–6.15)	4.74 (3.84–5.42)	0.001 *
HDL–C, mmol/L	1.19 (1.05–1.29) ^#^	1.44 (1.22–1.65) ^&^	1.15 (0.94–1.29)	0.001 *
LDL–C, mmol/L	1.96 (1.72–2.24) ^#&^	3.23 (2.69–3.84)	3.09 (2.46–3.68)	0.001 *
VLDL–C, mmol/L	0.37 ± 0.02 ^#&^	0.67 ± 0.05	0.54 ± 0.05	0.001 *
FBG, ×10^9^	5.73 ± 0.16	6.00 ± 0.34	5.663 ± 0.29	0.366
HbAlc, mmol/L	5.91 ± 0.09 ^&^	5.96 ± 0.16 ^&^	6.42 ± 0.23	0.010 *
HsCRP, mg/L	3.41 ± 1.57	1.81 ± 0.40	1.35 ± 0.24	0.996

WBC, white blood cells; HGB, hemoglobin; NEU, neutrophils; PLT, Platelets; UA, uric acid; CR, creatinine; Cys-C, cystatin C; ALT, alanine transaminase; ALB, albumin; DBIL, direct bilirubin; TG, triglycerides; CHOL, cholesterol; HDL-C, high-density lipoprotein cholesterol; LDL-C, low-density lipoprotein cholesterol; VLDL-C, very low-density lipoprotein cholesterol; FBG, fasting blood glucose; HbA1c, glycosylated hemoglobin; HsCRP, high-sensitivity C-reactive protein. Data are expressed as the mean ± SD. * *p* < 0.05, ^#^ *p* < 0.05 compare with the Partner group, ^&^ *p* < 0.05 compare with the Control group.

## Data Availability

Not applicable.

## Supplementary Materials

The following supporting information can be downloaded at: https://www.mdpi.com/article/10.3390/microorganisms10112288/s1, Table S1: The Framingham Stroke Profile.
