# Supplementary material for: The Spouses of Stroke Patients Have a Similar Oral Microbiome to Their Partners with an Elevated Risk of Stroke
Source: Microorganisms. 2022 Nov 18;10(11):2288. doi: 10.3390/microorganisms10112288 (PMC9697374; doi:10.3390/microorganisms10112288)
Supplement: Supplementary file 1 [file microorganisms-10-02288-s001.zip › microorganisms-2034837-supplementary.pdf]

Table S1 The Framingham Stroke Profile

	Points										
	0	+1	+2	+3	+4	+5	+6	+7	+8	+9	+10
Men											
Age,y	54-56	57-59	60-62	63-65	66-68	69-72	73-75	76-78	79-81	82-84	85
Untreated SBP, mm Hg	97-105	106-115	116-125	126-135	136-145	146-155	156-165	166-175	176-185	186-195	196-205
Treated SBP, mm Hg	97-105	106-112	113-117	118-123	124-129	130-135	136-142	143-150	151-161	162-176	177-205
Diabetes	No		Yes								
Cigarette smoking	No			Yes							
CYD	No				Yes						
AF	No				Yes						
LVH	No					Yes					

Points	10-Year Probability, %	Points	10-Year Probability, %	Points	10-Year Probability, %
1	3	11	11	21	42
2	3	12	13	22	47
3	4	13	15	23	52
4	4	14	17	24	57
5	5	15	20	25	63
6	5	16	22	26	68
7	6	17	26	27	74
8	7	18	29	28	79
9	8	19	33	29	84
10	10	20	37	30	88

	Points										
	0	+1	+2	+3	+4	+5	+6	+7	+8	+9	+10
Women											
Age,y	54-56	57-59	60-62	63-64	65-67	68-70	71-73	74-76	77-78	79-81	82-84
Untreated SBP, mm Hg		95-106	107-118	119-130	131-143	144-155	156-167	168-180	181-192	193-204	205-216
Treated SBP, mm Hg		95-106	107-113	114-119	120-125	126-131	132-139	140-148	149-160	161-204	205-216
Diabetes	No			Yes							
Cigarette smoking	No			Yes							
CYD	No		Yes								
AF	No				Yes						
LVH	No						Yes				

Points	10-Year Probability, %	Points	10-Year Probability, %	Points	10-Year Probability, %
1	1	11	8	21	43
2	1	12	9	22	50
3	2	13	11	23	57

4	2	14	13	24	64
5	2	15	16	25	71
6	3	16	19	26	78
7	4	17	23	27	84
8	4	18	27		
9	5	19	32		
10	6	20	37		

SBP indicates systolic blood pressure; CVD, cardiovascular disease, history of MI, angina pectoris, coronary insufficiency, intermittent claudication, or congestive heart failure; AF, atrial fibrillation; and LVH, left ventricular hypertrophy on ECG.

*The table gives the probability of stroke within 10 years for men and women 55–85 years of age and free of previous stroke in the Framingham Heart Study. To use these tables, identify each of the patient's characteristics and obtain the corresponding point value from the top row of the table. Sum points for each individual and then obtain corresponding 10-year probability of stroke. For example, a 64-year-old man (3 points) has a treated SBP of 138 mm Hg (6 points), no diabetes (0 points), does not smoke (0 points), or have CVD (0 points) or AF (0 points) but has LVH (5 points). His total point score (11 points) corresponds to an 11% 10-year probability of stroke.
